# Characterization of the Anti-Cancer Activity of the Probiotic Bacterium *Lactobacillus fermentum* Using 2D vs. 3D Culture in Colorectal Cancer Cells

**DOI:** 10.3390/biom9100557

**Published:** 2019-10-01

**Authors:** Joo-Eun Lee, Jina Lee, Ji Hyun Kim, Namki Cho, Sung Hoon Lee, Sung Bum Park, Byumseok Koh, Dukjin Kang, Seil Kim, Hee Min Yoo

**Affiliations:** 1 Stem Cell Research Center, Korea Research Institute of Bioscience and Biotechnology (KRIBB), Daejeon 34141, Korea; 2 Center for Bioanalysis, Korea Research Institute of Standards and Science (KRISS), Daejeon 34113, Korea; 3 College of Pharmacy and Research Institute of Drug Development, Chonnam National University, Gwangju 61186, Korea; 4 College of Pharmacy, Chung-Ang University, Seoul 06974, Korea; 5 Therapeutics & Biotechnology Division, Korea Research Institute of Chemical Technology (KRICT), Daejeon 34114, Korea; 6 Convergent Research Center for Emerging Virus Infection, Korea Research Institute of Chemical Technology, Daejeon 34114, Korea; 7 Department of Bio-Analysis Science, University of Science & Technology (UST), Daejeon 34113, Korea

**Keywords:** probiotics, *Lactobacillus fermentum*, colorectal cancer, apoptosis, spheroid, 3D culture

## Abstract

The aim of this study was to investigate the potential anti-cancer effects of probiotic cell-free supernatant (CFS) treatment using *Lactobacillus fermentum* for colorectal cancer (CRC) in 3D culture systems. Cell viability was assessed using MTS (3-(4,5-dimethylthiazol-2-yl)-5-(3-carboxymethoxyphenyl)-2-(4-sulfophenyl)-2*H*-tetrazolium, inner salt) assays, whereas apoptosis was monitored through RT-qPCR analysis of Bax, Bak, Noxa, and Bid mRNA expressions in addition to flow cytometry analysis of *Lactobacillus* cell-free supernatant (LCFS) treatment. Our results showed that the anti-cancer effect of LCFS on cell viability was pronouncedly enhanced in 3D-cultured HCT-116 cells, which was linked to the increased level of cleaved caspase 3. Additionally, upregulation of apoptotic marker gene mRNA transcription was dramatically increased in 3D cultured cells compared to 2D systems. In conclusion, this study suggests that LCFS enhances the activation of intrinsic apoptosis in HCT-116 cells and the potential anti-cancer effects of Lactobacilli mixtures in 3D culture systems. All in all, our study highlights the benefits of 3D culture models over 2D culture modeling in studying the anti-cancer effects of probiotics.

## 1. Introduction

Probiotics are important microorganisms in a healthy human microbiotic environment [1]. *Lactobacillus* and *Bifidobacterium* species are the most commonly used probiotics. Lactic acid bacteria (LAB) exert a number of health-promoting activities closely associated with the suppression of allergic responses as well as anti-inflammatory and anti-tumor effects [2,3,4,5]. The vast majority of studies on anticancer effects deal with colorectal cancer (CRC), which is the third most commonly occurring type of cancer worldwide [6]. Chemotherapeutic regimens including 5-fluorouracil (5-FU), oxaliplatin, and irinotecan, have been proven to be efficacious. In the case of metastatic CRC, the addition of targeted agents such as anti-EGFR monoclonal antibodies are considered [7,8]. Moreover, probiotics including *Lactobacillus* have been demonstrated to exhibit tumor-suppressive effects in colorectal cancer cell lines and in mouse tumor models [9]. Previous investigations have shown that *L. Casei* exerts anti-proliferative pro-apoptotic and anti-tumor effects in colon carcinoma cells [10]. Another group also showed that *L. Casei*-derived ferrichrome exerts a tumor-suppressive effect via the JNK signaling pathway [11]. According to another study, *Lactobacillus acidophilus* sensitizes colorectal cancer cells to 5-fluorouracil-induced apoptosis [12]. The aforementioned studies indicate that specific molecules secreted by probiotics cause anti-tumorigenic molecules to attack cancer cells [13].

In the past, most probiotic testing studies were performed using two-dimensional (2D) systems. However, 2D cultures are not able to completely recapitulate the three-dimensional (3D) interactions of cells and the extracellular matrix (ECM) within tissues [14]. Conversely, 3D cell cultures are better suited to restore intrinsic properties and imitate in vivo behavior compared to 2D cultures, which are monolayers on plastic. For example, a 3D culture of HNSCC cells has substantial differences from a 2D model in terms of drug response [15]. 3D models display augmented anti-tumor responses to AKT–mTOR–S6K and mitogen-activated protein kinase (MAPK) pathway inhibition compared to 2D models [16]. Comparative proteomic analysis of 2D- and 3D-cultured SW480 cells showed that XAV939, a poly-(ADP-ribosyl) transferase tankyrase inhibitor, suppresses the growth of SW480 cells in 3D cultures, but not in 2D cultures [17]. As such, 3D culture techniques have benefits for testing the effects of probiotics on cancer cells.

The aim of this study was to apply reliable in vitro 3D models with characteristics as similar as possible to in vivo cancer. Therefore, we used CRC cell lines in mechanistic differences as well as differences in probiotic cell-free supernatant (CFS) treatment response rate between 2D and 3D cultures.

## 2. Materials and Methods

### 2.1. Bacterial Cultures

*L. fermentum* was purchased from the Korean Collection for Type Cultures (KCTC 3112, Daejeon, Republic of Korea). Bacterial cultures were maintained through continuous subculturing in Lactobacilli De Man, Rogosa, Sharpe (MRS) broth (BD Difco Laboratories, Detroit, MI, USA). For the monitoring of bacteria growth, a wavelength of 620 nm was used to measure optical density (OD) LAMBDA UV/Vis Spectrophotometers (Perkin Elmer, Waltham, MA, USA).

### 2.2. Mammalian Cell Cultures

CCD18-Co, HCT-116, and HT-29 cell lines were purchased from the American Type Cell Collection (ATCC, Manassas, VA, USA). Cells were maintained in Dulbecco’s modified Eagle’s medium (DMEM) or Roswell Park Memorial Institute (RPMI) 1640 supplemented with 10% fetal bovine serum (FBS), 100 U/mL penicillin, and 100 μg/mL streptomycin. Phosphate-buffered saline (PBS), DMEM, RPMI, and FBS were purchased from Thermo Fisher Scientific. The cell lines were grown in a humidified 37 °C incubator with 5% CO_2_.

### 2.3. Spheroid Preparation

Three-dimensional (3D) cancer models were generated by seeding 6000 to 10,000 cells/well in ultra-low attachment (ULA) 96-well round bottom microplates and ULA 6-well flat bottom plates (Corning, Tewksbury, MA, USA). Multicellular cancer spheroids were obtained after the aggregation and compact clumping of cells. The spheroid was cultured for one, three, and seven days under standard culture conditions [18].

### 2.4. Preparation of Lactobacillus Cell-Free Supernatant (LCFS)

The bacteria were first grown and expanded in MRS broth. A stock solution was generated and stored in a deep freezer. *L. fermentum* was pelleted for 15 min at 1000× g. The bacteria were then resuspended in DMEM and cultured overnight at 37 °C, 5% CO_2_. To separate the bacterial pellets and conditioned media, the mixture was centrifuged for 15 min at 1000× g. The conditioned medium sample was filtered using 0.22 µm low protein binding cellulose acetate filters (Millipore, Billerica, MA, USA) and stored at −80 °C until use.

### 2.5. Cell Viability Assay

The antiproliferative effect of LCFS on colon cells was measured using the CellTiter 96 AQueous One Solution Cell Proliferation Assay Kit (Promega, Madison, WI, USA), following the manufacturer’s protocol. The cells were cultured at a density of 50–60% confluence and stabilized for 24 h (37 °C, 5% CO_2_). After LCFS treatment for 24 h, 48 h, and 72 h, 20 μL of CellTiter 96 AQueous One Solution Cell Proliferation Assay reagent was used for 30min–2 h at 37 °C. The signal was detected using an EnSpire Multimode Plate Reader (Perkin Elmer, Waltham, MA, USA).

### 2.6. Microscopic Analysis

The morphological changes of LCFS-treated cells were observed under a phase-contrast microscope (Olympus, Tokyo, Japan). After 24 h, 48 h, and 72 h of treatment with LCFS, images of the cells were taken (at 200× magnification) and alterations in morphology including cell size and shape were analyzed.

### 2.7. Apoptosis Assay

Cells treated with LCFS were detected via flow cytometry using the PE Annexin V Apoptosis Detection Kit (BioLegend, San Diego, CA, USA). LCFS-treated cells and untreated cells were incubated at 37 °C for 72 h. Each cell condition was washed with PBS and resuspended in Annexin binding buffer and stained with PE-conjugated Annexin V and 7AAD as per the manufacturer’s instructions. After staining, the cells were analyzed using a BD FACSVerse (BD Biosciences, San Diego, CA, USA). Data acquisition was conducted using the FlowJo software (TreeStar, Ashland, OR, USA) to investigate the percent of cells.

### 2.8. Western Blot

Cells treated with LCFS for 48 h were collected and lysed via a lysis buffer containing 25 mM of Tris-HCl (pH 7.6), 1% NP-40, 150 mM of NaCl, 1% sodium deoxycholate, 0.1% sodium dodecyl sulfate, and a cOmplete protease inhibitor cocktail (Roche Molecular Biochemicals, Basel, Switzerland). Lysates were clarified via centrifugation at 13,000 rpm at 4 °C for 10 min and the concentrations were measured using the Pierce BCA protein assay (Thermo Fisher Scientific, Waltham, MA, USA). Total protein was separated on an SDS page (NuPage, Bis-Tris Gel 4-12%; Thermo Fisher Scientific). Afterward, the gels were transferred to Polyvinylidene difluoride (PVDF) membranes (0.45 μM pore size) and the membranes were blocked with 5% nonfat dry milk. The primary antibody was diluted, according to the manufacturer’s recommendation: anti-BCL-2, anti-BAX, I-kappa-B-alpha (IκBα), phosphorylated I-kappa-B-alpha (p-IκBα), anti-actin (Santa Cruz Biotechnology, Dallas, TX, USA), and anti-cleaved caspase 3 (Cell Signaling Technology, Danvers, MA, USA). After this process, the membranes were incubated with secondary antibodies (Jackson Laboratory, Bar Harbor, ME, USA) at 1:5000 dilutions, which were detected with an ImageQuant LAS 4000 mini (Fujifilm, Tokyo, Japan). For the quantification of the western blot, the band was determined using an image analysis program (Multi Gauge Ver. 3.0, Fujifilm, Tokyo, Japan).

### 2.9. RT-PCR

Total RNA was isolated from probiotic supernatant-treated cells for 48 h using the RNeasy mini kit (QIAGEN, Hilden, Germany). Each PCR reaction was performed as a 20 µL reaction containing iTaq™ Universal SYBR^®^ Green Supermix (Bio-Rad, Hercules, CA, USA). The comparative Ct method was applied to complete the fold-difference in the expression levels relative to a control sample. The RT-qPCR analysis was implemented on a StepOnePlus Real-Time PCR system (Thermo Fisher Scientific, Waltham, MA, USA).

### 2.10. Confocal Imaging

Cells treated with LCFS for 48 h were fixed in 4% paraformaldehyde. Fixed cells were blocked and permeabilized with 5% BSA and 0.05% Triton X-100 in PBS. Primary antibody used was cleaved caspase-3 (Cell Signaling Technology) and incubated overnight at 4 °C. The secondary antibody used was donkey anti-rabbit Alexa Fluor 555 (Thermo Fisher Scientific). Antibodies were used 1:100 concentrations, binding overnight at 4 °C. DNA was stained with DAPI. The images were acquired using a LSM 800 confocal microscope with Zeiss software.

### 2.11. Statistical Analysis

Statistical analysis was performed using GraphPad Prism 5 (GraphPad Software, Inc., San Diego, CA, USA) and the values were given in mean ± SEM. The statistical results were further analyzed using the unpaired t-test. P-values less than 0.05 were considered as statistically significant.

## 3. Results

### 3.1. L. fermentum Cell-Free Supernatants (LCFS) Promote Cell Death in 3D HCT-116 Conditions rather than in 2D HCT-116 Conditions

**Figure 1:**
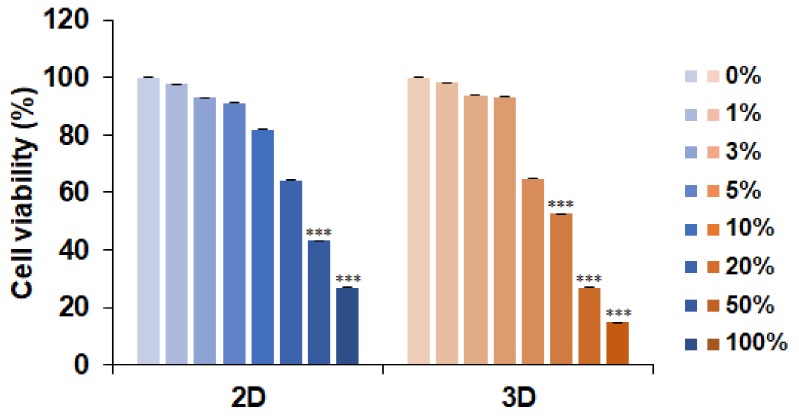
Antiproliferative effects of *L. fermentum* Cell-Free Supernatants (LCFS) on 2D and 3D HCT-116 cells. Cell viability was determined using the MTS assay 72 h after treatment with LCFS (*n* = 3, *** *p* ≤ 0.001).

**Figure 2:**
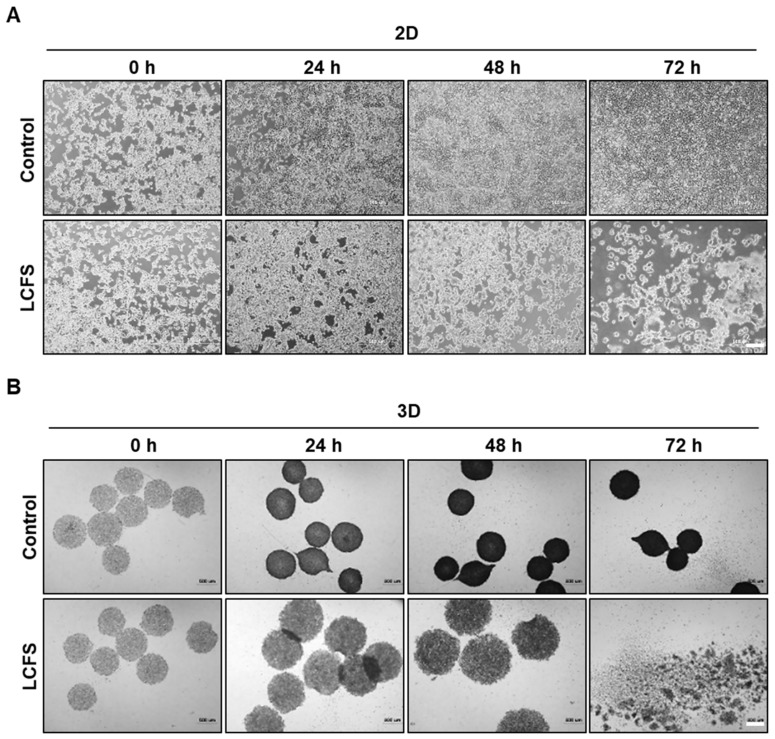
Anti-cancer effect of LCFS in 2D and 3D HCT-116 colon cancer cells. (**A**) Morphological changes 2D HCT-116 cells after 24 h, 48 h, and 72 h of treatment with LCFS. A significant increase in detachment cells was observed after treatment over different time periods (*n* = 3, magnification 4× magnification, scale bar: 500 μm). (**B**) Analysis of spheroid formation on 96-well plates. HCT-116 cells were grown as spheroids (6000 cells/well) and treated with LCFS for 24 h, 48 h, and 72 h. Apoptotic morphology increased after treatment with LCFS (*n* = 3, 4× magnification, scale bar: 500 μm).

To examine the anti-cancer effects of LCFS and investigate the anti-cancer effects of LCFS in a 2D and 3D culture system, we generated 3D tumor spheroids. Cells of the HCT-116 colon cancer cell line were analyzed for spheroid formation capacity in an ultra-low attachment dish. The HCT-116 cells were seeded (6000 cells/well) into 6-well and 96-well ultra-low attachment plates and cultured at standard conditions to acquire spheroids. LCFS was screened against 2D and 3D HCT-116 colon cancer cell lines using a cell proliferation assay (CellTiter Clo) (Figure 1). The viability of colon cancer cell monolayers was reduced to 50% after LCFS treatment. Colon cancer spheroids treated with 20% LCFS resulted in the reduced viability of the colon cancer spheroids. LCFS showed stronger cytotoxicity against HCT-116 in 3D conditions. Another colorectal cancer cell line, HT-29, also showed similar results in terms of its viability (Figure S1B). Interestingly, the viability of LCFS-treated CCD18-Co, which is a normal colorectal cell, did not lead to cell death (Figure S1A). Next, the 2D and 3D HCT-116 cells were exposed to LCFS for 0, 24, 48, and 72 h (Figure 2A,B). Under the LCFS conditions, a diffuse outer layer of cells appeared at 48 h and the shape of the spheroids was completely lost after 72 h (Figure 2B). The results indicate that LCFS possesses anti-cancer effects and stronger cytotoxicity against 3D spheroids than monolayer colon cancer cell lines.

### 3.2. LCFS Induces Apoptosis of HCT-116 Spheroids

**Figure 3:**
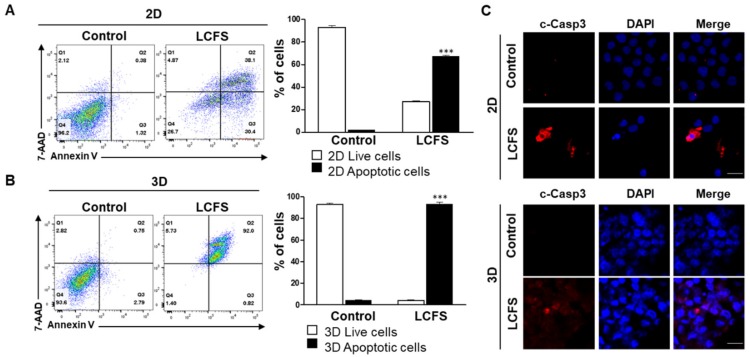
Determination of LCFS-increased apoptosis in 2D and 3D HCT-116 cells. (**A**) Apoptosis determined through flow cytometry. The results show that LCFS could induce apoptosis in 2D and 3D HCT-116 (*n* = 3, *** *p* ≤ 0.001, 100× magnification, scale bar: 20 μm). (**B**) Apoptosis analysis was performed in 3D HCT-116 using flow cytometry (*n* = 3, *** *p* ≤ 0.001, 100× magnification, scale bar: 20 μm). (**C**) 2D and 3D of LCFS-treated cells were determined using confocal imaging with antibodies specific for cleaved caspase 3 proteins.

To examine whether LCFS-induced cell death was related to apoptosis, we performed Annexin V-PE and 7-AAD double staining followed by fluorescence-activated cell sorting. LCFS-treated 2D and 3D HCT-116 and HT-29 colon cancer cell lines showed an increase in the number of apoptotic cells within 72 h after treatment. However, in the absence of the drug treatment group, the apoptotic cells were reversed (Figure 3A,B and Figure S2A,B). Using confocal microscopy, we discovered that LCFS increased cleaved caspase 3 in 3D spheroid conditions rather than in 2D monolayers after 72 h (Figure 3C). The data indicate that LCFS promotes apoptosis in spheroids and has potential as an anti-colon cancer drug that has benefits over 2D monolayers.

### 3.3. LCFS Promotes Apoptotic Markers in Spheroids More Sensitive than in Monolayers

**Figure 4:**
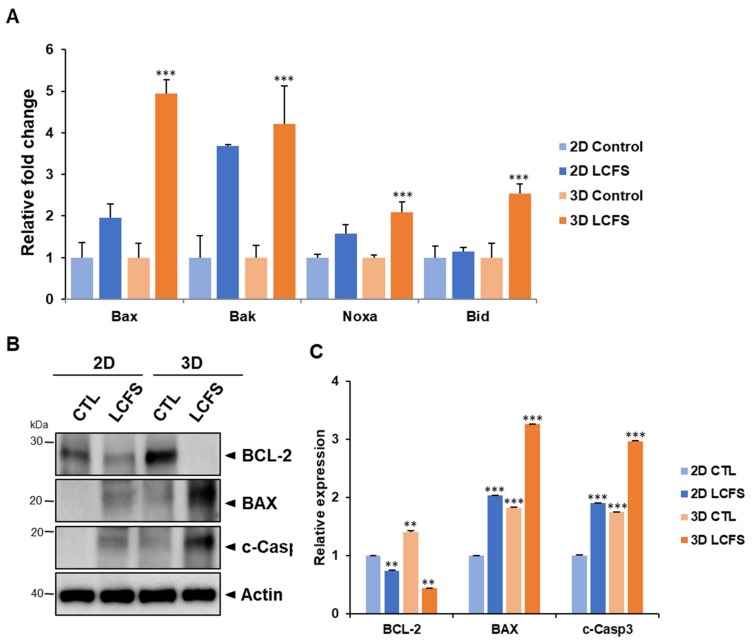
LCFS-induced apoptosis markers in 2D and 3D colon cancer cells. (**A**) Comparison of the qRT-PCR analysis results of the mRNA levels of Bax, Bak, Noxa, and Bid of the control and the LCFS-treated 2D and 3D HCT-116 cells (*n* = 3, ** *p* ≤ 0.01 *** *p* ≤ 0.001). (**B**) Whole cell lysates from LCFS-treated cells were immunoblotted with antibodies specific for BCL-2, BAX, and cleaved caspase 3 proteins. (**C**) Bar graph for BCL-2, BAX, and cleaved caspase 3 ratio (*n* = 3, ** *p* ≤ 0.01 *** *p* ≤ 0.001).

**Figure 5:**
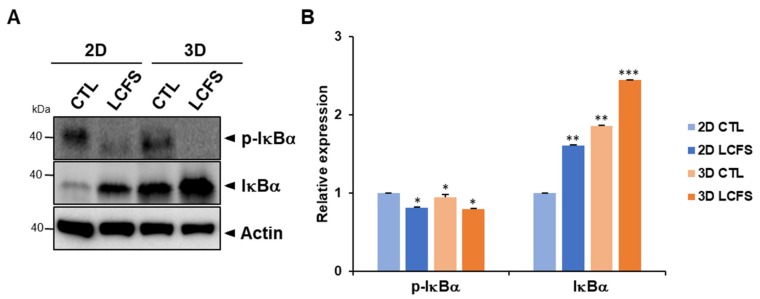
Determination of the LCFS-increased apoptosis mechanism in 2D and 3D HCT-116 cells. (**A**) Whole cell lysates were used to determine the expression levels of I-kappa-B-alpha (IκBα) and p-IκBα after treating cells with LCFS. (**B**) Bar graph for of I-kappa-B-alpha (IκBα) and p-IκBα ratio (*n* = 3, * *p* ≤ 0.05 ** *p* ≤ 0.01 *** *p* ≤ 0.001).

The qRT-PCR results demonstrated that LCFS increased pro-apoptotic markers such as Bax, Bak, Noxa, and Bid, which promoted apoptosis in both 2D and 3D-cultured HCT-116 cells compared to untreated control cells (Figure 4A). Moreover, qRT-PCR analysis revealed that the LCFS treatment condition significantly increased the mRNA expression level of pro-apoptotic markers in 3D spheroids to a greater degree when compared to 2D. Moreover, we determined apoptotic proteins in HCT-116 and HT-29 cell lines as LCFS downregulated the expression of the antiapoptotic protein BCL-2 (a survival factor in apoptosis regulation) and upregulated BAX and cleaved caspase 3 (Figure 4B C, and Figure S3A,B). In addition, LCFS induced cell apoptosis in HCT-116 and HT-29 cells through the downregulation of p-IκBα, which is related to NF-κB signaling, a critical transcription factor that regulates multiple genes associated with tumorigenesis (Figure 5A,B, Figure S3C,D). The data indicate that LCFS promotes apoptosis and has potential as an anti-colon cancer drug that targets 3D spheroids.

## 4. Discussion

3D culture systems can be used for the investigation of new predictive markers and mechanisms for cancer treatment. In the past, the in vitro tumor suppressive effect of culture supernatants derived from probiotics were tested using two-dimensional (2D) cultures [19]. However, 3D spheroids have the benefits of better mimicking in vivo tumor environments compared to 2D cultures [20,21]. Furthermore, there is a need to establish trustworthy methods for the measurement of cell proliferation, viability, and death for cells growing in spheroids.

Several studies have been performed using 2D models of cancer cell lines to investigate the causes of different phenotype and genetic alterations when exposed to various CFS treatments. Although some 2D CRC cell culture models have been published, such models generally use commercially available cell lines such as the Caco-2, HT-29, FaDu, SW-480, or HCT-116 CRC cell lines [2,22]. For several decades, research in this field has been focused on both the influence of pathogenic bacteria on CRC and the potential of beneficial bacteria to suppress cancer growth. The literature regarding the involvement of *L. fermentum* in apoptotic cell death is still limited. Previous studies have shown that the protective effect of autophagy activated by *L. fermentum* in acetaminophen (APAP) induced cytotoxicity in HepG2 cells [23]. *L. fermentum* RM28 also triggered the antiproliferation of colon cancer cells [24]. The significant antioxidant, antiproliferative, and pro-apoptotic activities of *L. fermentum* on CRC cells have been shown to have beneficial properties in attenuating the risk of CRC development [3]. However, all of the aforementioned findings were obtained using 2D culture systems; therefore, further experiments should be conducted using 3D culture systems.

Apoptosis can be induced by activating intrinsic or extrinsic pathways. The intrinsic pathway involves pro-apoptotic proteins, Bax, Bak, Noxa, and Bid, and promotes its release from mitochondria and apoptosome formation. The extrinsic pathway involves death receptors, death-inducing signaling complexes, and caspase activation [25,26]. Our results demonstrate the potential of *L. fermentum* cell-free supernatants to induce the apoptotic cell death of CRC in both 2D and 3D. The mRNA levels of apoptosis markers involved in intrinsic pathways are induced to a greater degree after LCFS exposure in 3D conditions when compared to 2D. However, increased levels of BCL-2 and less cleaved-caspase 3 were expressed in the 3D spheroid control compared to 2D cultures in Figure 4B and less NF-κB activation was observed in 3D cultures compared to 2D cultures in Figure 5A. The advantages of culturing cells in 3D involve elevating cell–cell interactions to mimic in vivo systems and inducing the generation of a hypoxic core. Inner zones contain quiescent and hypoxic cells and hypoxic regions that trigger the expression of cytoprotective proteins that allow for survival and proliferation [27,28]. Moreover, abnormal expressions of Bcl-2 are commonly found in cancer cells, which increase the chemoresistance of these cells [29,30]. Similar to human tumors, proliferating, quiescent, and dying cells coexist in normoxic, hypoxic, or necrotic zones within spheroids [31,32,33].

Previous studies have investigated immunomodulating effects using heat-killed bacteria and cell-free supernatants. The data indicate that heat-killed bacteria and their fractions (cell-free supernatant, CFS) and purified components have key probiotic effects, with advantages over live probiotics in terms of safety [34]. We propose that CFS is a suitable alternative strategy for testing probiotic effects on 3D spheroids. Further studies should be conducted to test whether heat-killed bacteria or purified components have similar effects on 3D culture systems.

Current 2D cell cultures cannot mimic the advantages of 3D such as the heterogeneity and complexity of biochemical and signaling molecules, which are critical for cell physiology [31,35]. In addition, the loss of tissue-specific properties is common in 2D monolayer models. As a result, researchers have identified solid tumor growth in 3D, resulting in heterozygous exposure to oxygen as well as physical and chemical stresses [36,37,38,39,40]. Overall, a uniform-sized 3D tumor spheroid platform could be a powerful tool for anti-cancer drug discovery and the development process of such drugs using materials from probiotics such as CFS and bacteria itself [36,41,42].

## 5. Conclusions

Our study demonstrated the anti-cancer effect in 3D-cultured HCT-116 cells activated by CFS originating from *L. fermentum*. The mechanism of action of LCFS in colon cell lines using 3D systems has never been reported in previous studies. In this study, we showed that LCFS inhibits cell growth by inducing apoptosis in colorectal cell lines to exert its antiproliferative activity by preventing NF-κB signaling. We were able to demonstrate that LCFS can become a potent multitarget anti-cancer chemotherapeutic agent that induces cell death. The findings could serve as a notable reference for the testing of cell-free supernatants from probiotics for the purpose of killing tumors, although safety, side effects, and anti-tumor efficacy should be further tested using 3D platforms.

## Supplementary Materials

The following are available online at https://www.mdpi.com/2218-273X/9/10/557/s1, Figure S1: Effect of LCFS on the viability of colon cancer cells and normal cells in 2D and 3D. (A) Normal cells (CCD-18Co) were treated with various concentrations. (B) Likewise, colon cancer cells (HT-29) were incubated with LCFS under the same conditions. Cell viability was determined using the MTS assay 72 h after treatment with LCFS (*n* = 3, *** *p* ≤ 0.001); Figure S2: Determination of LCFS-increased apoptosis in 2D and 3D HT-29 cells. Apoptosis determined with a (A) 2D model and (B) 3D model of HT-29. Apoptosis was assessed through flow cytometry. The results show that LCFS can induce apoptosis in 2D and 3D HT-29 (*n* = 3, *** *p* ≤ 0.001); Figure S3: Determination of the LCFS-increased apoptosis markers and mechanisms in the 2D and 3D colon cancer cells. (A) Whole cell lysates from LCFS-treated HT-29 cells were immunoblotted with antibodies specific for BCL-2, BAX, and cleaved caspase 3 proteins. (B) Bar graph for BCL-2, BAX, and cleaved caspase 3 ratio (*n* = 3, ** *p* ≤ 0.01 *** *p* ≤ 0.001). (C) Whole cell lysates from LCFS-treated HT-29 cells were used to determine the expression levels of I-kappa-B-alpha (IκBα) and p-IκBα after treating cells with LCFS. (D) Bar graph for of I-kappa-B-alpha (IκBα) and p-IκBα ratio (*n* = 3, * *p* ≤ 0.05 ** *p* ≤ 0.01 *** *p* ≤ 0.001).
