# Supplementary material for: Characterization of the Anti-Cancer Activity of the Probiotic Bacterium Lactobacillus fermentum Using 2D vs. 3D Culture in Colorectal Cancer Cells
Source: Biomolecules. 2019 Oct 1;9(10):557. doi: 10.3390/biom9100557 (PMC6843223; doi:10.3390/biom9100557)
Supplement: Supplementary file 1 [file biomolecules-09-00557-s001.pdf]

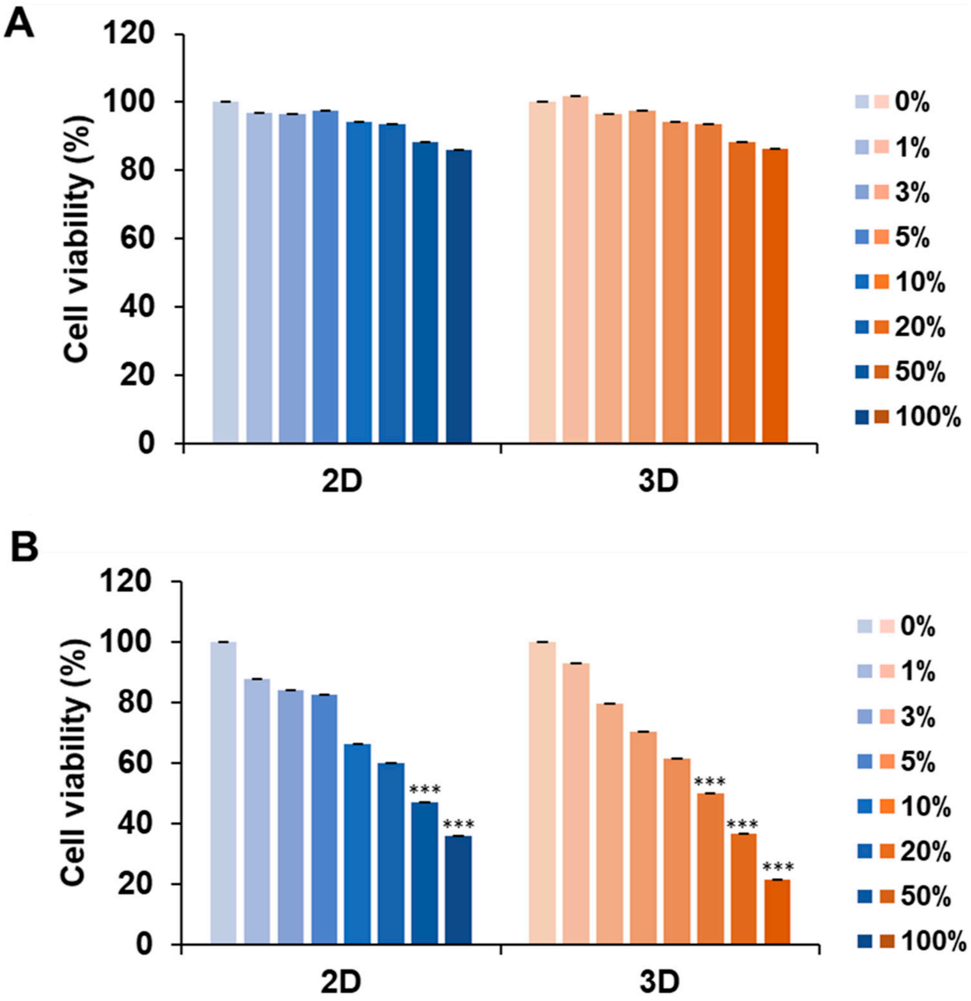

Figure S1. Effect of *Lactobacillus* cell-free supernatant (LCFS) on the viability of colon cancer cells and normal cells in 2D and 3D. (**A**) Normal cells (CCD-18Co) were treated with various concentrations. (**B**) Likewise, colon cancer cells (HT-29) were incubated with LCFS under the same conditions. Cell viability was determined using the MTS (3-(4,5-dimethylthiazol-2-yl)-5-(3-carboxymethoxyphenyl)-2-(4-sulfophenyl)-2H-tetrazolium, inner salt) assay 72 h after treatment with LCFS ($n = 3$, *** $p \leq 0.001$)

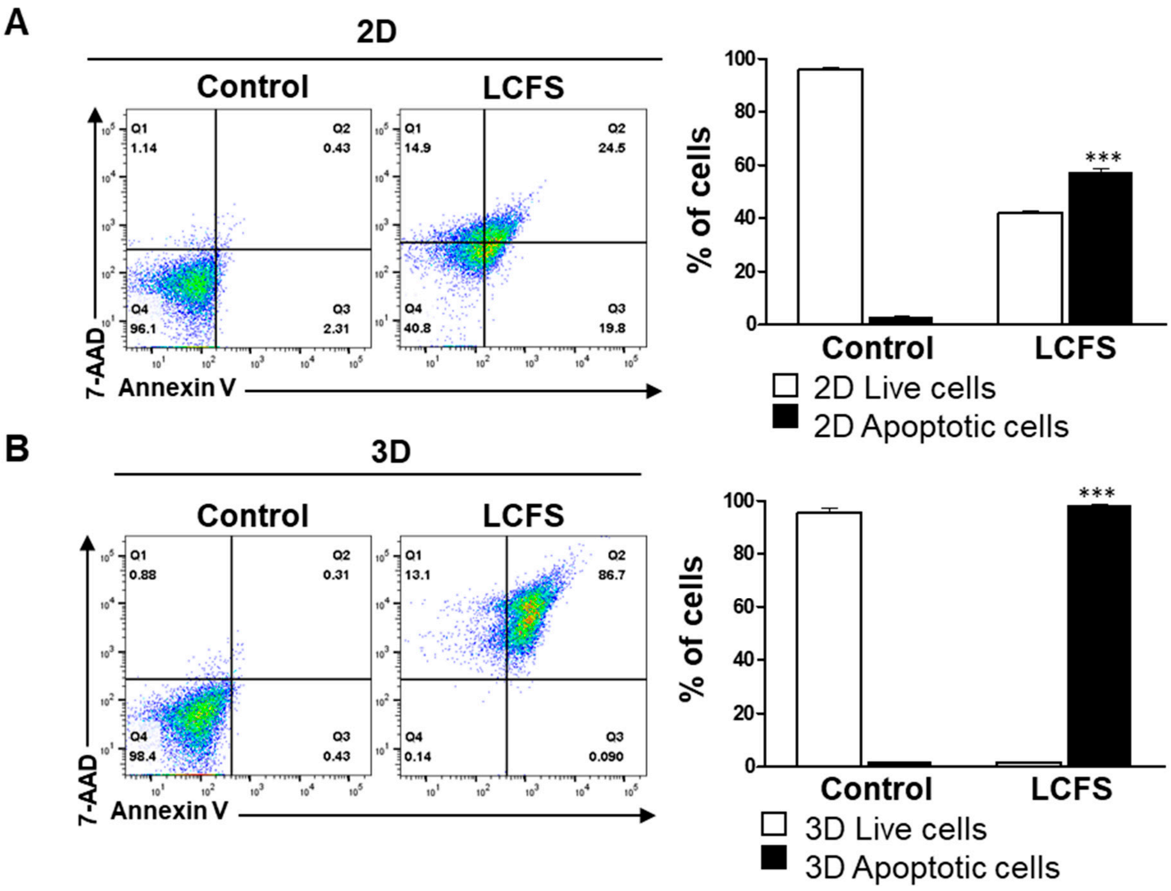

Figure S2. Determination of LCFS-increased apoptosis in 2D and 3D HT-29 cells. Apoptosis determined with a (**A**) 2D model and (**B**) 3D model of HT-29. Apoptosis was assessed through flow cytometry. The results showed that LCFS could induce apoptosis in 2D and 3D HT-29 ($n = 3$, *** $p \leq 0.001$).

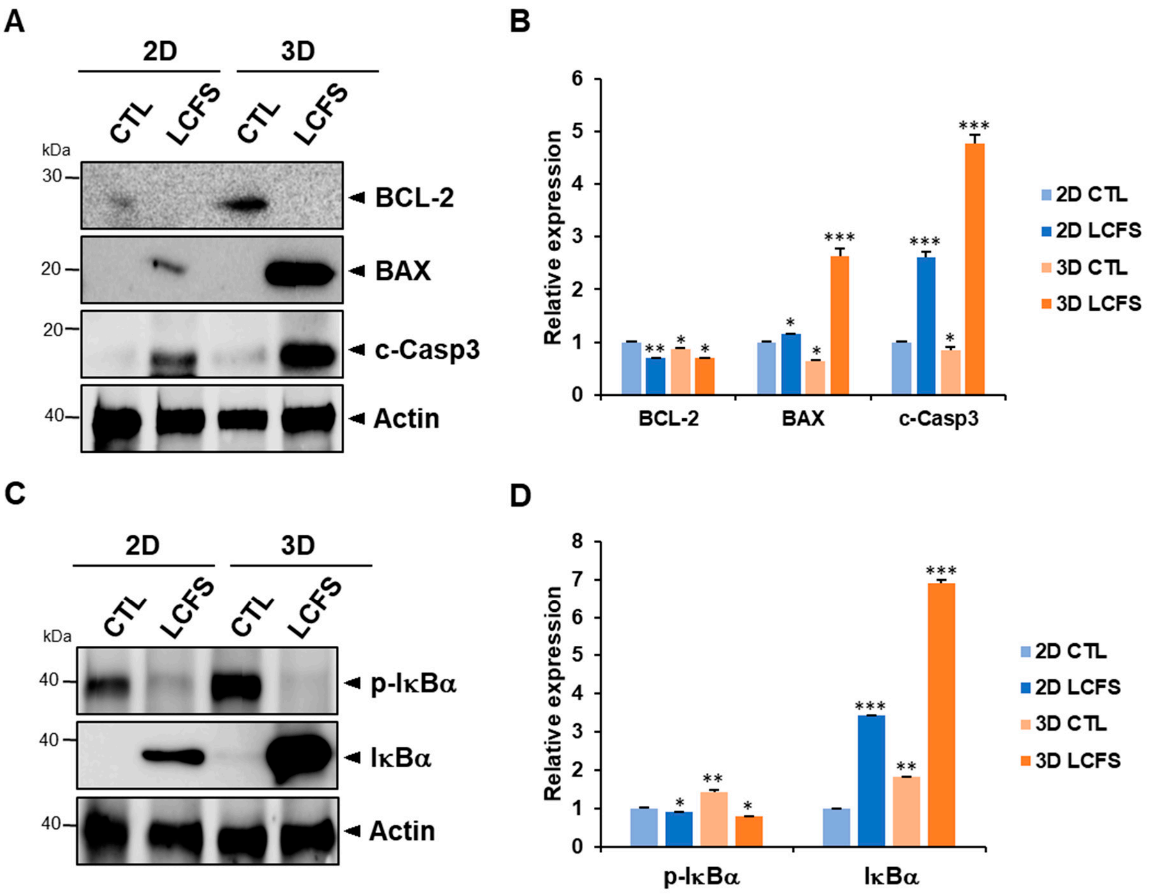

Figure S3. Determination of the LCFS-increased apoptosis markers and mechanisms in 2D and 3D colon cancer cells. (**A**) Whole cell lysates from LCFS-treated HT-29 cells were immunoblotted with antibodies specific for BCL-2, BAX, and cleaved caspase 3 proteins. (**B**) Bar graph for BCL-2, BAX, and cleaved caspase 3 ratio (n = 3, ** $p \leq 0.01$ *** $p \leq 0.001$). (**C**) Whole cell lysates from LCFS-treated HT-29 cells were used to determine the expression levels of I-kappa-B-alpha (IκBα) and p-IκBα after treating cells with LCFS. (**D**) Bar graph for of I-kappa-B-alpha (IκBα) and p-IκBα ratio (n = 3, * $p \leq 0.05$ ** $p \leq 0.01$ *** $p \leq 0.001$).
